# Pb_x_(OH)_y_ cluster formation and anomalous thermal behaviour in STI framework-type zeolites

**DOI:** 10.1038/s41598-022-20317-1

**Published:** 2022-09-24

**Authors:** Georgia Cametti, Diana P. Roos, Damien Prieur, Andreas C. Scheinost, Sergey V. Churakov

**Affiliations:** 1 Institute of Geological Sciences, Bern University, Baltzerstrasse 1+3, 3012 Bern, Switzerland; 2 The Rossendorf Beamline at the European Synchrotron Radiation Facility (ESRF), Avenue des Martyrs 71, 38043 Grenoble, France; 3 Institute of Resource Ecology, Helmholtz Zentrum Dresden Rossendorf, Bautzner Landstrasse 400, 01328 Dresden, Germany; 4 Paul Scherrer Institut, Forschungstrasse 111, 5232 Villingen PSI, Switzerland

**Keywords:** Materials science, Mineralogy

## Abstract

For the first time, the structural investigation of a Pb-exchanged zeolite (Pb_13.4_(OH)_10_Al_17.4_Si_54.6_O_144_ ∙38H_2_O) with **STI** framework type, revealed a highly unusual and intriguing sudden volume increase under continuous heating. Understanding the fundamental mechanisms leading to such an unusual behaviour is essential for technological applications and interpretation of chemical bonding in zeolites. The dehydration was tracked in situ from 25 to 450 °C by single crystal X-ray diffraction, infrared and X-ray absorption spectroscopy. Further interpretation of the experimental observations was supported by ab initio molecular dynamics simulations. Initially, Pb-STI unit-cell volume contracts (ΔV = − 3.5%) from 25 to 100 °C. This agrees with the trend observed in **STI** zeolites. Surprisingly, at 125 °C, the framework expanded (ΔV =  + 2%), adopting a configuration, which resembles that of the room temperature structure. Upon heating, the structure loses H_2_O but no de-hydroxylation occurred. The key mechanism leading to the sudden volume increase was found to be the formation of Pb_x_(OH)_y_ clusters, which prevent the shrinking of the channels, rupture of the tetrahedral bonds and occlusion of the pores. This zeolite has therefore an increased thermal stability with respect to other **STI** metal-exchanged zeolites, with important consequences on its applications.

## Introduction

Zeolites are an important class of crystalline porous materials, which occur in nature and are extensively synthesized in laboratory. Thanks to their specific properties, they find applications in several fields like petro-chemical industry, pollution control, environmental remediation, energy storage, etc.^1,2^.

The practical use of zeolites depends on numerous factors, which are mainly related to their porous structure. The latter consists of a three-dimensional interconnected framework of tetrahedra (TO_4_) (T mainly Si,Al), which form cavities and channels where a large variety of extraframework (EF) species and H_2_O are host^3,4^.

The porous structure is very responsive to changes in temperature and water vapour pressure and can, therefore, easily release or absorb H_2_O. Of particular interest are the structural modifications occurring as a function of increasing temperature since many zeolites find use only after thermal treatment^5,6^. The extent of such modifications can vary from minor to severe. For instance, some zeolites experience upon dehydration the rupture of the tetrahedral bonds T-O-T of the framework^7^ with subsequent collapse of the structure. These changes influence the microporous properties and therefore the performance of the material. Thus, their understanding and ultimately the prediction of the thermal stability is of paramount importance.

In contrast to dense solid phases, most zeolites undergo a negative thermal expansion (NTE) and typically their volume contracts with increasing temperature^6^. This process is caused by the release of the structural H_2_O and the subsequent migration of the EF cations toward the wall of the framework in order to reach a more balanced coordination environment. In contrast, positive thermal expansion has been rarely observed^8^. It must be noticed that a slight expansion of the unit-cell volume at the early stage of heating is commonly due to weakening of hydrogen bonds, but not to H_2_O loss.

In general, the extent of the transformations and therefore the modification of the structural topologies upon heating, depend on the framework type and the kind of EF species that occupy the pores^6,7^.

In previous studies, we reported the effect of heavy-metals incorporation on the thermal stability of a microporous zeolite with **STI** framework type^9–11^. The latter has symmetry *Fmmm* that can be lowered depending on the type and distribution of the extraframework occupants. The topology at room temperature (phase A) consists of two systems of interconnected channels: The first is composed by 8-membered rings of tetrahedra, running parallel to [001], and the second is formed by ten-membered rings and runs parallel to [100]^12^. The interconnection of tetrahedra originates the t-sti-1* cavity^13^, where the EF cations and H_2_O are hosted. Zeolites with **STI** framework type experience upon heating severe framework modifications, which lead to: (i) the rupture of specific T-O-T connections of the framework; (ii) the formation of new structural topologies (namely phase B^14–17^, B’^11^, D^18^, D’^10^) and, (iii) the occlusion of the ten-membered ring channels with subsequent loss of porosity^9,10,18^. The aforementioned structural changes lead to significant contraction of the pores accompanied by decrease of the unit-cell volume.

**Figure 1:**
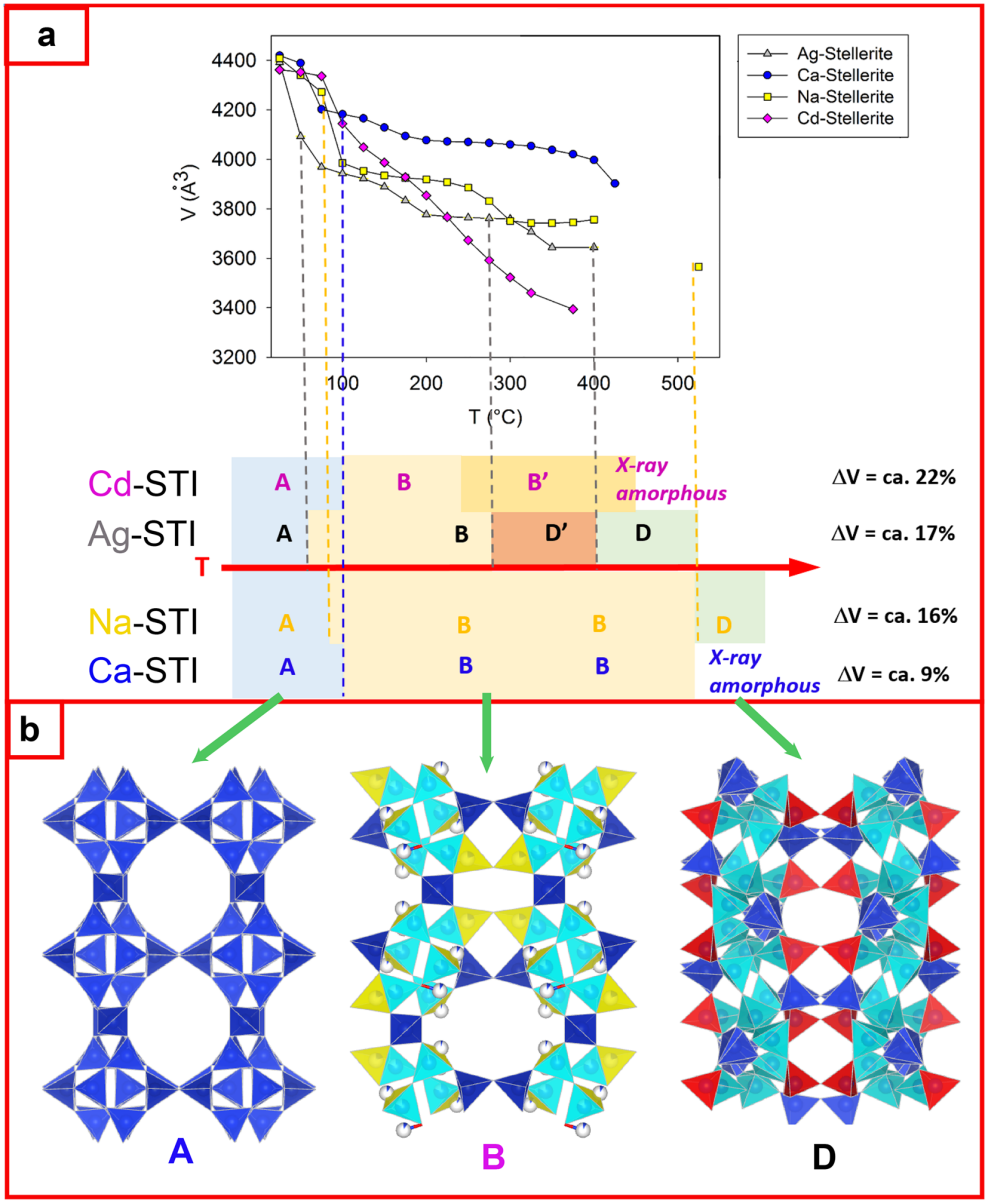
Typical temperature-dependent behavior and structural transformations observed in zeolites with **STI** framework type, containing different EF cations. (**a**) The graph reports the unit-cell volume as a function of increasing temperature for Ag^10^-, Ca^9^-, Na^9^-, and Cd^11^-stellerite obtained by single crystal X-ray diffraction. The capital letters (A, B, B’, D’, D) refer to the name of the corresponding phases observed as a function of increasing temperature for each metal-exchanged form. The contraction of the unit-cell volume (ΔV) is reported as a percentage with respect to the one measured at room temperature. (**b**) Representative structures for the A, B, and D-phase topology. The aluminosilicate framework is shown as blue tetrahedra. Cyan, yellow and red tetrahedra represent those tetrahedra involved in the T-O-T breaking process for each phase.

The extraframework cations in **STI**-type zeolites (Fig. 1) influence: (1) the structural modifications (i.e. phase B, B’, D, D’) that form upon heating, (2) the unit-cell volume trend, and (3) the upper temperature limit of the structural stability.

In this study, we report on the behaviour of Pb-exchanged stellerite (Pb-STI) upon heating. We monitor in situ the structural changes with increasing temperature and we demonstrate that the dehydration behaviour as well as the associate structural transformations are anomalous compared to, not only those of **STI** framework type zeolites, but also to the commonly reported unit-cell volume decrease observed in zeolitic materials. To the best of our knowledge, it is the first time that a sudden volume increase at elevated temperature, is reported for a zeolite structure. This increase is accompanied by a reversal of the structural transformations, which finally result in a high-temperature phase with the same framework configuration as the RT structure. We will show that this phenomenon can be explained by the formation of Pb_x_(OH)_y_ clusters during the dehydration process. Most important, it leads to increased thermal stability and has therefore important consequences for the applications of these materials.

## Results

### Structural modifications upon heating

**Figure 2:**
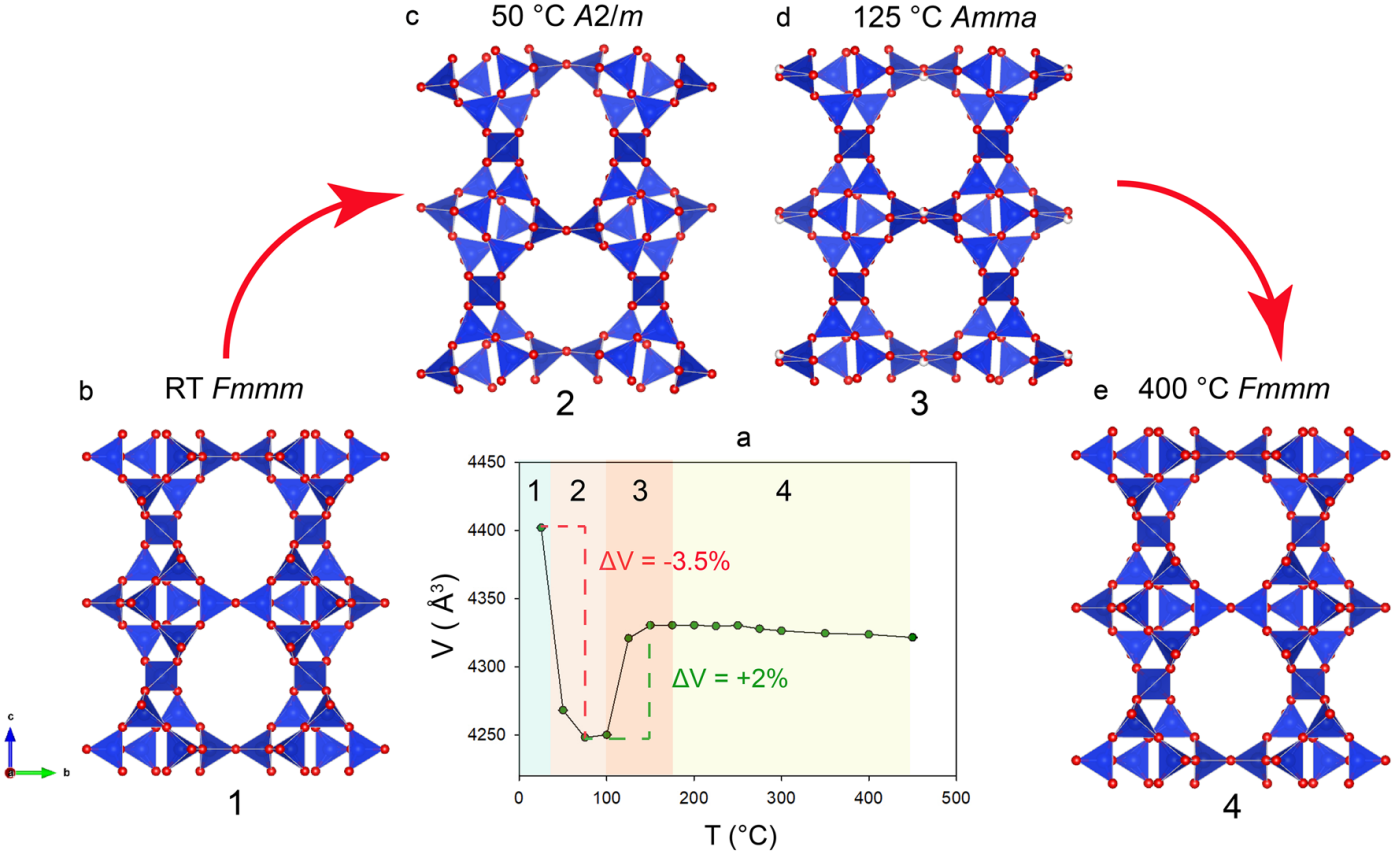
(**a**) Evolution of the unit-cell volume of Pb-STI as a function of temperature obtained by SC-XRD. (**b**–**e**) Corresponding structural changes in **STI** framework of Pb-stellerite observed in situ at different temperatures.

The unit-cell volume of Pb-STI, with corresponding framework modifications, was determined by in situ Single Crystal X-ray Diffraction (SC-XRD) from room temperature (RT) to 450 °C (Fig. 2). Three different transformation stages can be distinguished:

- From RT to 100 °C, the unit-cell volume gradually decreases by 3.5% with respect to that measured at RT^19^. This change agrees with the common trend observed for **STI** framework type zeolites (Fig. 1) and can be associated with an initial dehydration process^9–11^. The structural transformation in this temperature range (Fig. 2a–c) is characterized by the shrinking and deformation of the ten-membered ring channels parallel to [100] that become more elliptical. This structural modification is at 50 °C accompanied, by the lowering of the crystal symmetry from *Fmmm* to *A*2/*m* (Table 1).
- From 100 to 150 °C, in contrast to the common behaviours of **STI** type zeolites, the unit-cell volume increases by approximately 2%. The space group at 125 °C turns into orthorhombic (space group *Amma*) and the channels expand, adopting the original roundish shape, characteristic to the RT structure (Table 1, Fig. 2d).
- From 175 to 450 °C, the unit-cell volume does not vary significantly. The structure gradually transforms from *Amma* to *Fmmm* space group (Table 1, Fig. 2e).

**Table 1:** Crystal data and refinement parameters of Pb-STI at 50, 125, and 400 °C.

Crystal data	PbSTI 50	PbSTI 125	PbSTI 400
*a* (Å)	13.6431(3)	13.6684(3)	13.6261(4)
*b* (Å)	17.8035(5)	17.8690(4)	17.9186(5)
*c* (Å)	17.5725(4)	17.6896(4)	17.7090(5)
*β *(°)	89.977(2)	90	90
*V* (Å^3^)	4268.27(18)	4320.52(17)	4323.8(2)
*Z*	1	1	1
Space group	*A*2*/m*	*Amma*	*Fmmm*
Refined chemical formula	Pb_11.2_(OH)_8_(Si,Al)_72_O_144_∙ 20.3H_2_O	Pb_11.42_(OH)_8_(Si,Al)_72_O_144_∙ 15.9H_2_O	Pb_11.41_(OH)_9.9_(Si,Al)_72_O_144_
Crystal size (mm)	0.07 × 0.100 × 0.200	0.07 × 0.100 × 0.200	0.07 × 0.100 × 0.200
**Intensity measurement**			
Diffractometer	Bruker Apex II	Bruker Apex II	Bruker Apex II
X-ray radiation	Mo*Kα* λ = 0.71073 Å	Mo*Kα* λ = 0.71073 Å	Mo*Kα* λ = 0.71073 Å
X-ray power	50 kV, 60 mA	50 kV, 60 mA	50 kV, 60 mA
Monochromator	Graphite	Graphite	Graphite
Temperature (°C)	50	125	400
Exposure time (s)	10	10	10
Max. 2θ (°)	53.31	51.36	54.34
Index ranges	− 17 ≤ *h* ≤ 17	− 14 ≤ *h* ≤ 16	− 17 ≤ *h* ≤ 14
	− 22 ≤ *k* ≤ 22	− 21 ≤ *k* ≤ 21	− 22 ≤ *k* ≤ 21
	− 22 ≤ *l* ≤ 21	− 21 ≤ *l* ≤ 20	− 22 ≤ *l* ≤ 22
No. of measured reflections	27,181	20,759	11,097
No. of unique reflections	4536	2218	1330
No. of observed reflections *I* > 2σ (I)	3169	1506	1074
**Structure refinement**			
No. of parameters used in the refinement	399	209	121
*R*(int)	0.0824	0.0693	0.0444
*R*(σ)	0.0806	0.0522	0.0275
GooF	1.100	1.035	1.055
*R*1, *I* > 2σ (*I*)	0.1019	0.0931	0.0607
*R*1, all data	0.1378	0.1277	0.0749
*wR*2 (on *F*^2^)	0.2498	0.2647	0.1614
Δρ_min_ (− eÅ^−3^) close to	− 1.48 C1	− 1.28 C7	− 1.03 C1A
Δρ_max_ (eÅ^−3^) close to	1.33 C9A	1.15 C6	0.83 C1B

Thus, the thermal behaviour of Pb-STI is characterized by an initial volume contraction and a subsequent expansion, which leads to a structural topology (phase A) equal to that observed at RT. This mechanism is anomalous not only for **STI** framework-type zeolites, but in general for zeotype materials, which are normally characterized by a negative thermal expansion. Most impressive is the sudden volume increase at a given temperature accompanied by the expansion of the contracted framework, which is usually observed only after rehydration. Moreover, the thermogravimetric analysis^19^ indicated a gradual and continuous release of (most probably) H_2_O upon heating that should be associated with a channel contraction and corresponding decrease of the unit-cell volume.

**Figure 3:**
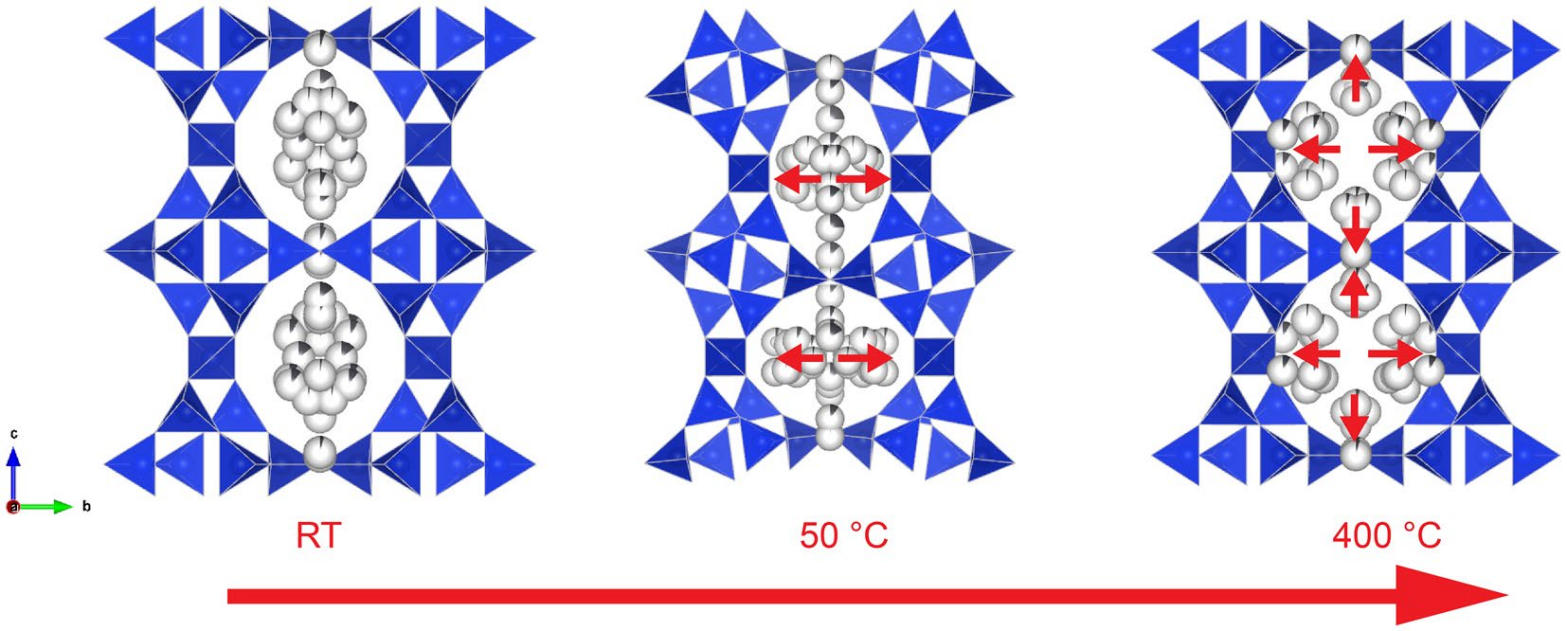
Polyhedral representation of the Pb-STI structure at RT, 50 °C and 400 °C, refined from SC-XRD data. Partially colored dark-grey spheres represent partially occupied Pb sites. Red arrows indicate the direction of EF cations movement upon heating. For sake of clarity, only the Pb sites are shown.

Because of the strong disorder of the EF occupants (Pb^2+^, H_2_O and OH^−^), their positions as well as the eventual diffusion and release of the EF species could not be unequivocally monitored by SC-XRD. From the structural refinements, it was possible, however, to follow the variation of the electron-density distribution associated with the EF content. The latter was refined as several low occupied sites, close to each other. At RT, the EF species were mainly concentrated at the centre of the ten-membered ring channels and in the middle of the eight-membered ring window between two t-sti-1* cavities^19^. With the increase of temperature and subsequent shrinking of these channels, Pb and eventually OH groups came closer to the wall of the framework. When the structure expanded and the channels adopted again a roundish configuration, the EF species remained close to the framework wall (Fig. 3).

### Dehydration of the structure

**Figure 4:**
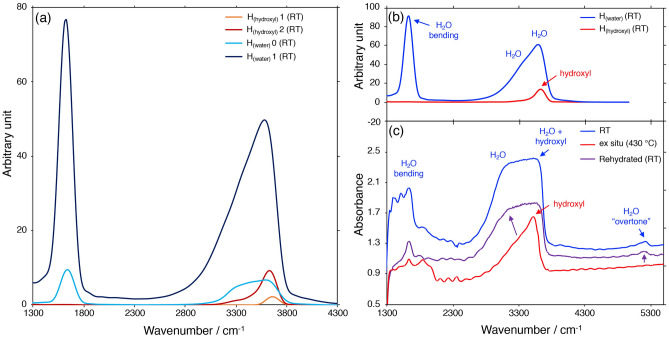
(**a**) VDOS curves calculated for hydrogen belonging to OH^−^ bonded to 1 Pb (orange), hydrogen belonging to OH^−^ bonded to 2 Pb (red), hydrogen belonging to “free” H_2_O molecules (light blue) and hydrogen belonging to H_2_O bonded to 1 Pb (dark blue), based on the theoretical structural model of Pb-STI at RT^19^. (**b**) VDOS curves calculated for hydrogen belonging to H_2_O (blue) and hydrogen belonging to OH^−^ groups (red). (**c**) Selected IR absorption spectra of Pb-STI at RT (blue), after ex situ thermal treatment (red) and after rehydration (purple).

Spectroscopic analyses were performed to obtain more information about the dehydration process. A distinction between H_2_O and OH^−^ by infrared (IR) analysis was complicated by the fact that these molecules vibrate at similar frequencies. Vibrational Density of States (VDOS) curves were calculated for hydrogen atoms (H) based on the DFT-equilibrated Pb-stellerite structure at room temperature from our previous study^19^. Different bands in the IR spectra were assigned to specific molecular groups (Supplementary material S1): vibrations of H_2_O are found as a sharp peak between 1500 and 1800 cm^−1^ and a broader peak between 3000 and 3800 cm^−1^, while only one rather sharp vibrational peak of OH^−^ is found between 3500 and 3800 cm^−1^ (Fig. 4a,b).

Experimentally measured IR absorption spectra of Pb-stellerite are reported in Fig. 4c and Fig. S1. The region of the H_2_O bending vibrations (1500–1800 cm^−1^) is strongly influenced by the background measurements due to thickness variations in the quartz glass capillary (quartz glass has an IR absorption peak at 1635 cm^−1^) and was therefore not included in the interpretation. Instead, another absorption peak from 5100 to 5300 cm^−1^ was detected in the spectrum that results from the bend and stretch combination mode of the H_2_O molecule^20^. This peak is present in the spectra obtained at RT (Fig. 4c, Fig. S1a), indicating that the structure is hydrated.

IR spectra collected after the ex situ thermal treatment up to 430 °C are displayed in Fig. 4c, Fig. S1b. The peak between 5100 and 5300 cm^−1^, as well as the left shoulder of the broad, flat absorption band between ca. 2700 and 3700 observed at RT, disappeared. The only remaining absorption band in the spectra after the thermal treatment is sharp and at ca. 3500 cm^−1^, similar to the calculated VDOS curve for H belonging to OH^−^ groups (Fig. 4b). Thus, it can be concluded that Pb-STI, at high temperatures, contains OH^−^ groups but is H_2_O free.

### Reversibility of the dehydration process

After the ex situ thermal treatment, Pb-STI crystals were exposed to high humidity conditions (relative humidity > 90%) in order to allow reabsorption of H_2_O. IR absorption curves of the crystals obtained after 15 days equilibration time are shown in Fig. 4c, Fig. S1c. The absorption on the left shoulder of the broad peak between 2700 and 3700 cm^−1^ increased and so did the absorption of the H_2_O bend and stretch combination peak, illustrated by purple-coloured arrows in Fig. 4c. This indicates that the structure of Pb-STI is able to reabsorb H_2_O and that the dehydration process is reversible.

The reversibility of the dehydration process was also investigated by SC-XRD. After an equilibration time of 15 days under high humidity conditions (relative humidity > 90%), new data were collected at RT on a previously dehydrated crystal. The rehydrated structure has *Fmmm* symmetry and lattice parameters a = 13.6119(7), b = 18.1834(9), c = 17.8460 (10) Å and V = 4417.1(4) Å^3^. The latter are very similar to those of the RT structure. The deviation of the unit cell volume with respect to the RT Pb-STI is smaller than 0.34%. The positions of the EF sites do not differ from those in RT Pb-STI^19^, despite the disorder (Fig. S2, Table S1).

The pronounced similarity between RT Pb-STI and rehydrated Pb-STI in terms of both structural and spectroscopy analyses consistently indicates that Pb-STI de- and rehydration as well as its structural modifications upon heating are reversible processes.

### Structural models of the high temperature phase

The experimental results showed that the fully exchanged Pb-STI has a higher thermal stability with respect to the pristine material (Ca-STI^9^) and the other metal-exchanged forms (Na-STI^9^, Ag-STI^10^, Cd-STI^11^). In particular, no formation of the structural modifications B, B’, C and D, D’ occurs upon heating and the framework does not experience the breaking of the tetrahedral bonds T-O-T. Consequently, it does not lose its microporous structure at high temperatures. The most surprising observation is the expansion of the zeolitic framework after the initial contraction, which is induced by dehydration. According to TGA^19^ and IR analysis, H_2_O is lost as a function of increasing temperature whereas, as demonstrated by IR spectra collected at higher temperatures (430 °C), the OH^−^ groups are retained in the dehydrated structure.

The interpretation of this unique behaviour was complicated by the disorder of the EF species. Thus, different hypotheses were tested by means of MD simulations. A detailed description of the set up and the length of trajectories for each structural model are given in the Methods session.

According to SC-XRD data, two main structural configurations form upon heating: (i) a contracted **STI** topology, with space group *A*2/*m* characterized by elliptical channels, and (ii) an “expanded” structure, compared to that observed at 75 °C, with *Fmmm* space group and roundish channels, which resemble those of the RT phase.

The first structural configuration was well reproduced by the theoretical model containing 12 Pb p.f.u. and 0.8 H_2_O per Pb, simulated at 75 °C (Pb12-75-10 W) (Fig. S3, Table S2). Thus, we can assume that at 75 °C, Pb-STI structure is partially dehydrated.

To reveal the mechanism, which brings to the formation of the second structural topology, and to understand the evolution of the EF species upon heating, three potential scenarios (M1, M2, M3) were considered:

- M1: Dehydration of the structure without any further reaction involved. In this case, the reference model consisted of a fully dehydrated Pb-STI (Pb_12_OH_8_Si_56_Al_16_O_144_).
- M2: Partial H_2_O dissociation at elevated temperatures. Dissociation of H_2_O molecules based on the hydrolysis reaction H_2_O ⇌ OH^−^ + H^+^ has been reported in literature for zeolite L^21^.
- M3: Oxidation of some Pb^2+^ ions to Pb^4+^. The reaction Pb^2+^  + 2 H_2_O ⇌ Pb^4+^  + 2OH^−^ + H_2(g)_ was hypothesized for Pb-exchanged zeolite A treated at high temperatures^22^. The produced OH^−^ groups coordinate Pb and the H_2_, a gas, is assumed to leave the crystal structure.

The analysis of the tested models showed that the dehydrated Pb-STI (M1) has an extremely contracted unit- cell volume with the resulting structural configuration similar to that of the other dehydrated forms of **STI** zeolites^9–11^ (Fig. S4a, Table S3). Therefore, this model was not further considered. The “hydrolysis” hypothesis (M2) was ruled out based on the obtained equilibrated structures (Fig. S4b, Table S3), the configuration of which has also contracted channels, not in agreement with the experimental structure measured at 400 °C. Moreover, 60% of OH^−^ groups re-protonated, indicating that the deprotonated form is less stable than the H_2_O molecules. The theoretical model that better matched the experimental data, in terms of unit-cell volume and framework configuration, was the M3 scenario, i.e. a Pb-STI with 17% oxidized Pb^4+^ (Fig. S4c, Table S3). However, XANES spectra collected in situ as a function of temperature, did not show any evidence of a change of the oxidation state of Pb^2+^ (Fig. S4d), not supporting this hypothesis.

### Dehydrated Pb-STI with Pb_x_(OH)_y_ clusters

**Figure 5:**
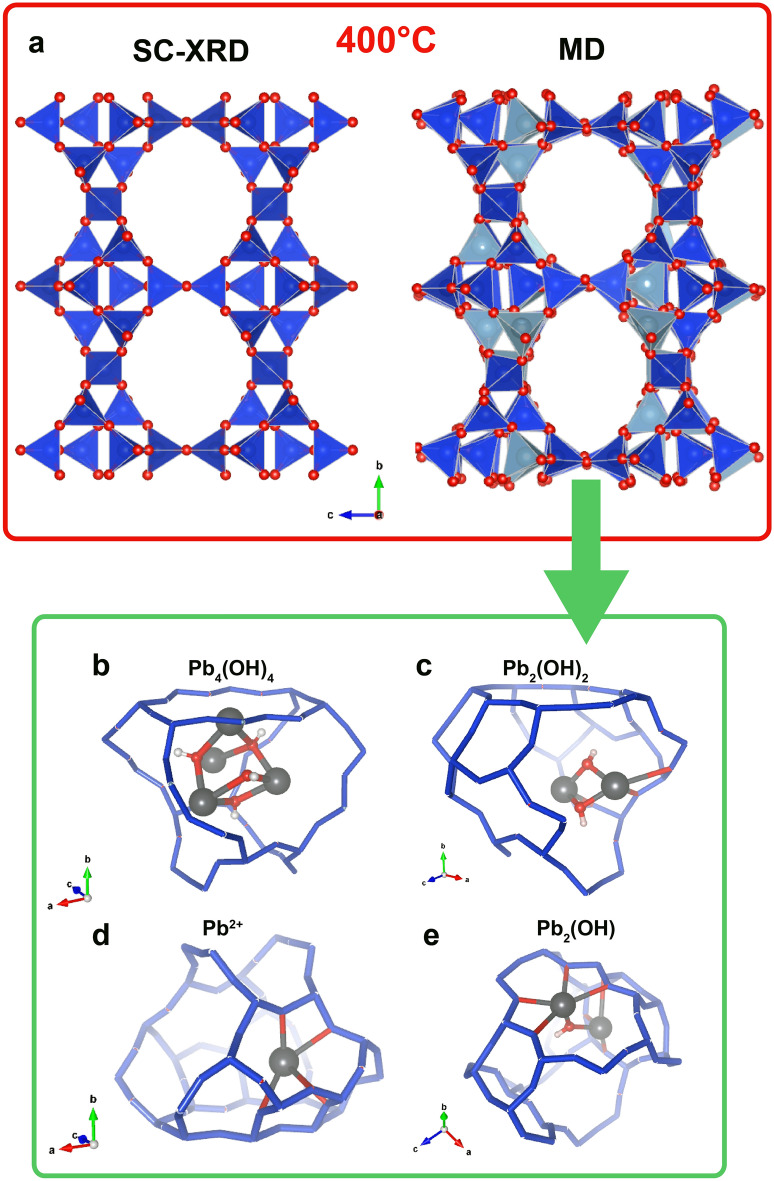
(**a**) Comparison between the experimental structure measured at 400 °C by SC-XRD and the simulated one corresponding to the model Pb12-400-C4. The framework is displayed by polyhedral representation. (**b**–**e**) Fragment of the simulated structure (average atomic coordinates) showing the different arrangement of PbOH species in the t-sti-1* cavities of Pb-STI. Dark grey spheres represent Pb atoms. Oxygen and hydrogen are depicted as red and white spheres, respectively. The framework is shown as blue sticks.

Finally, a series of structural models (M4) containing different Pb_x_O_y_ and Pb_x_(OH)_y_ clusters (1 < x,y < 4) was tested (Table 2). Among the structural configurations, the one that better reproduced the experimental data was a dehydrated model with a composition Pb_12_OH_8_Si_56_Al_16_O_144_ where 33% and 16% of Pb formed Pb_4_OH_4_ and Pb_2_OH_2_, respectively (reference model name Pb12-400-C4). The framework of the equilibrated structure well reproduced that of the refined Pb-stellerite at 400 °C, characterized by the roundish channels (Fig. 5a) parallel to [100]. The size of the horizontal and vertical maximum length was 7.25 and 8.68 Å, respectively, in good agreement with the experimental values (7.01 and 8.99 Å).

**Table 2:** Summary of the structural models containing different kind of clusters simulated by MD.

Reference name	M4		
	Pb12-400-C4O	Pb12-400-C2	Pb12-400-C4
Supercell content	24 Pb, 304 O, 16 H, 112 Si, 32 Al	24 Pb, 304 O, 16 H, 112 Si, 32 Al	24 Pb, 304 O, 16 H, 112 Si, 32 Al
Temperature	400 °C	400 °C	400 °C
Number of clusters type	2 Pb_4_(OH)_2_O_2_ 2 Pb_2_O_2_	4 Pb_2_O_2_	2 Pb_4_(OH)_4_ 2 Pb_2_(OH)_2_
pre-equ | equ t (ps)	8 |8	4| 5	12 | 13
*a* (Å)	13.676	13.652	13.645
*b* (Å)	17.493	17.570	17.783
*c* (Å)	17.719	17.747	17.966
*V* (Å^3^)	4237	4255	4358
α	80.74	90.05	89.69
β	90.10	89.72	89.86
γ	89.24	89.71	89.58

The resulting average unit-cell parameters are reported for each model.

The simulation suggests the existence of different [Pb_x_(OH)_z_]^y+^ complexes in the zeolitic cavities: i) [Pb_x_(OH)_x_]^y+^ (x = 2, 4) clusters in the middle of the cavities; ii) as single cation Pb^2+^ bonded to the oxygen-atoms of the framework, and iii) as [Pb_2_(OH)]^3+^ species bonded to the oxygen atoms of the framework. The Pb atoms in the Pb_4_(OH)_4_ clusters adopt an almost tetrahedral shape (Fig. 5b) with average Pb-OH bond distances equal to 2.35 Å. Pb atoms are on average at 3.82 Å far from each other, with the shortest Pb-Pb contact found at 3.64 Å. Similarly, the Pb_2_(OH)_2_ clusters are found close to the wall of the framework, where they bond with the tetrahedral oxygen atoms (Fig. 5c). The corresponding Pb-OH (average = 2.27 Å) and Pb–O distances (average = 2.63 Å) are shorter than those in Pb_4_(OH)_4_.

The single Pb^2+^ cations are mainly found at the wall of the six-membered ring apertures of the t-sti-1* cavity, with average Pb–O distances = 2.5 Å (Fig. 5d). Finally, the [Pb_2_(OH)]^+3^ species consist of two Pb atoms, which share an OH group (with average Pb-OH distances = 2.26 Å) and bond to further oxygen atoms of the framework at longer distances (Pb–O = 2.64 Å) (Fig. 5e).

## Discussion

Our structural characterisation of Pb-STI reveals for the first time a highly unusual thermal behaviour in a microporous zeolite. The mechanism is unique in two ways:

- The sudden volume increase at ca. 100 °C is accompanied by the reversal of the structural modification. The structure transforms back to its original framework configuration. Commonly, a slight expansion of the unit-cell volume is observed in some zeolites, but only after complete dehydration, before the structural collapse^23,24^. Such minor volume changes do not lead however to the transformation back to the RT structure, unless rehydration occurs.
- The expansion apparently prevents an immediate structural collapse commonly observed in zeolites upon dehydration. The thermal stability of Pb-STI increased significantly with respect the other **STI** zeolites. The diffraction data collected at high temperature indicate good crystallinity of the sample up to 450 °C.

This unprecedented behaviour is of high relevance because Pb-STI is the only exchanged form maintaining the RT framework configuration (and therefore its microporous properties) up to 450 °C. To the best of our knowledge, this is the first time that such an anomalous thermal behaviour is reported in zeolites.

The sudden increase of the unit-cell volume is associated with the formation of Pb(OH) clusters, which prevent the further shrinking of the channels. The MD simulations demonstrated that a dehydrated Pb-STI without clustering of Pb^2+^ and OH^−^, results in a contracted topology. Therefore, the formation of Pb_4_(OH)_4_ clusters must be the key mechanism, which leads to the channel expansion between 125 and 150 °C.

The unique dehydration behaviour of Pb-STI can be explained according to the following process: at first, the water loss is accompanied by the unit-cell volume contraction, in line with the general negative thermal expansion observed in zeolitic materials. At 75 °C, the structure is partially hydrated and between 100 and 125 °C, in correspondence of the increase of the unit-cell volume, the cluster formation starts. Residual water might be retained up to 250–270 °C, and in a subsequent step completely released, as indicated by the slight unit-cell volume decrease between 250 and 450 °C and by TGA curve^19^ (Fig. S5). The different thermal behavior of STI zeolites exchanged with different EF cations (Ca, Na, Ag, Cd) was related not only to the size and charge of the counter cation^9–11^, but also to the electronic structure and strength of chemical bonding of the EF cations with H_2_O and framework oxygen atoms^11^. In light of the results obtained on Pb-STI, we can conclude that the nature of the cationic species (cation, hydroxyl, etc.) which are up taken during the exchange process as well those forming upon heating, have an impact on the dehydration path. In Pb-STI, the clustering of Pb(OH)^+^ species during the dehydration prevents the collapse of the structure because no enough space is left in the channels to accommodate the “flipping” of the tetrahedra, which would be involved in the breaking of T–O–T bonds, typical of zeolites with **STI** framework type.

The formation of metal clusters in zeolites under thermal treatment was at first hypothesized by Uytterhoeven et al.^25^. The authors stated that, “H_2_O are dissociated by the strong electrostatic fields of the Me^2+^ ions” leading to the formation of metal clusters of the type Me^+^–O–Me^+^ or Me^+^–OH. Baekelant et al.^26^ used the same hypothesis to explain the behaviour of Pb-LTA zeolite upon heating. They reported the formation of Pb_4_(OH_2_)_x_ (x = 2–4) and subsequently, of Pb_4_O_z_ (z = 2–4) clusters upon increasing temperature, suggesting that the OH^−^ groups dehydroxylate and transform to extraframework oxygen. Pb_4_(O,OH)_4_ clusters were also found in the mineral maricopaite, the first natural zeolite with Pb as a dominant extraframework cation^27^. This natural zeolite contains, at room temperature, H_2_O and OH^−^ groups. Pb atoms form Pb_4_ tetrahedra capped by OH or H_2_O. At date, no investigation of its dehydration behaviour has been conducted.

In our study, the process leading to the clusters formation is different from that described by Uytterhoeven et al.^25^ and Baekelant et al.^26^ for Pb-LTA zeolite. The Pb_x_OH_x_ clusters do not form because of water dissociation, since the OH species are already present in the hydrated structure at RT, i.e. the **STI** zeolite takes up Pb in form of Pb^2+^ and Pb(OH)^+^ species^19^ during the exchange procedure. In addition, no dehydroxylation takes place up to 450 °C, as demonstrated by the IR spectra of the thermally treated sample. The latter process would indeed imply the presence of protons attached to framework oxygen atoms. Last hypothesis was tested by the MD simulations (Pb12-400-C2, Pb12-400-C4O) and could be rejected on the basis of the obtained results.

These results suggest that the incorporation of Pb in form of (PbOH)^+^ species into zeolites can increase the thermal stability and widen the range of temperatures for which the structural changes are reversible. Further studies must be conducted to ascertain if this unusual trend can be generalized to all Pb-zeolites containing Pb(OH)^+^ and H_2_O at RT, and if and how this mechanism might be tuned for specific purposes.

## Methods

### Sample

The sample under investigation was a Pb-stellerite (Pb-STI) produced and characterized at room temperature in our previous study^19^. This Pb-STI was obtained by cation exchange of a natural stellerite^28^, previously exchanged with Na (Na-STI), in 0.5 M Pb-acetate solution for 4 weeks at 95 °C. The electron microprobe analyses coupled with X-ray Absorption Spectroscopy^19^ indicated the presence of [Pb(OH)]^n+^ species within the channels and resulted in the chemical composition: Pb_13.4_(OH)_10_Al_17.4_Si_54.6_O_144_ ∙38H_2_O.

### In situ high-temperature single crystal X-ray diffraction

A single crystal with dimensions 0.070 × 0.100 × 0.200 mm was glued on the tip of a glass fibre and mounted on a goniometer head. This was the same crystal used for the RT data collection in our previous study^19^. The diffraction data were collected on a Bruker APEX II diffractometer equipped with a CCD detector, MoKα fine focus tube and an in house N_2_ blower. The high temperature (HT) data were obtained by heating the crystal stepwise from 50 to 450 °C (maximum allowed temperature of our equipment), in steps of 25 °C. Before each data collection, the crystal was equilibrated for at least 30 min. The same experimental set up was used for the investigation of the thermal stability of the other cationic forms of stellerite (i.e. Ca-, Na-, Ag-, and Cd-STI)^9–11^ and is regarded as dry experimental conditions.

The data were integrated and corrected for absorption using the software package Apex3.v.2018.7-2^29^. Structure solution (ShelxS^30^) for the data set at 50, 75 and 100 °C pointed to the monoclinic space group *C*2/*m*, transformed to the non-standard setting *A*2/*m* for better comparison with literature data. From 125 to 150 °C, the structures were solved and refined (ShelxL^31^) in the *Amma* space group, and from 200 to 450 °C in *Fmmm*.

The reversibility of the dehydration process (rehydration) was also investigated by SC-XRD. Another Pb-STI crystal was selected, because the one used during the first set of high-temperature measurements was lost after the experiment. After a stepwise thermal treatment up to 200 °C for approximately 6 h, the crystal was exposed to high humidity conditions (relative humidity > 90%) for 15 days. Subsequently, diffraction data were collected at RT with data collection strategy similar to that used for the first crystal.

Crystal data and refined parameters of the Pb-STI measured at 50 (PbSTI 50), 125 (PbSTI 125), and 400 °C (PbSTI 400) are reported in Table 1.

Structural data of Pb-STI obtained at different temperatures have been deposited in form of Crystallographic Information File (Cif). All structural drawings were produced with the software VESTA^32^.

### Molecular dynamics simulations

Ab initio simulations were performed with the CP2K package^33^. The interatomic forces were obtained based on the Density Functional Theory (DFT)^34^. The electron exchange and correlation energies were approximated with the Perdew-Burke-Ernzerhof (PBE) functional^35^. Dispersion correction was applied using the semi-empirical pair potential type DFT-D3^36^. The Gaussian and plane waves basis sets were used to represent electron wave function and the electron density, respectively^37^. Valence electron wave functions were described by the Gaussian short range Double-Zeta Valence Polarized basis set (DZVP-MOLOPT)^38^. Core electrons were represented by dual space norm conserving pseudopotentials^39^. MD simulations were performed in an isothermal-isobaric (NPT) ensemble with a flexible cell. The temperature was controlled by the Canonical Sampling Velocity Rescaling (CSVR) thermostat^40^. The integration time step was set to 0.5 fs. Initial structures for MD simulations were obtained by geometry optimisation of the hypothetical structural models.

### Structural models

Different structural models of Pb-stellerite in terms of H_2_O-, Pb-content and temperature were tested. Here we report those that are discussed in the Results session.

All structures consisted of a fully flexible 2 × 1 × 1 Pb-STI supercell. The starting coordinates of the framework were taken from the refined structures obtained from SC-XRD data. The following configurations were considered:

- Partially dehydrated Pb-stellerite at 75 °C (Pb12-75-10 W) (Table S2); The initial coordinates were obtained starting from the equilibrated structure of RT Pb-stellerite, with chemical composition Pb_12_(OH)_8_Si_56_Al_16_O_144_ ∙34H_2_O^19^ by removing 14 H_2_O.
- Dehydrated Pb-stellerite at 400 °C (M1: Pb12-400-0W) (Table S3); The dehydrated structure, with chemical composition Pb_12_(OH)_8_Si_56_Al_16_O_144_ was simulated by using two different approaches. At first, the water was removed stepwise by increasing the temperature and the MD trajectories were run at 400 °C. A second approach consisted in using the atomic coordinates of the framework of the refined structure at 400 °C, and distributing the Pb and OH- groups as indicated by the electron density distribution of the XRD data, i.e. close to the wall of the framework. No significant differences were noticed between the two models; therefore, we reported here the results from the second approach.
- Pb-stellerite with hydrolysed water 400 °C (M2: Pb12-400-hy) (Table S3); This model had chemical composition Pb_12_(OH)_28_H_20_Si_56_Al_16_O_144_, and corresponds to a structure where 20 H_2_O (estimated as the number of H_2_O in the partially hydrated structure) underwent the hydrolysis reaction: H_2_O ⇌ OH^−^ + H^+^. The structure contains additional 20 OH^−^ groups p.f.u., bonded to Pb, and 20 H^+^ p.f.u., attached to framework oxygen atoms.
- Pb-stellerite with oxidized Pb^4+^ at 400 °C (M3: Pb12-400-10Pb4 and Pb12-400-4Pb4) (Table S3). In this case, the oxidation of Pb^2+^ to Pb^4+^ was hypothesized. Two MD simulations were run at 400 °C, one assuming 5 out of 12 Pb p.f.u. (42%) and the other one 2 out of 12 Pb p.f.u. (17%) in the oxidized form. Additional 10 OH^−^ groups p.f.u. were added to the structural model hypothesizing 42% of all Pb to be oxidized whereas 4 additional OH^−^ groups were added assuming an oxidation of 17% of all Pb ions.
- Dehydrated Pb-stellerite containing Pb_x_(OH)_y_ and Pb_x_O_y_ clusters, with initial values x = y = 2, 4 (M4: Pb12-400-Cx) (Table 2). The clusters were distributed in the t-sti-1* cavities and the remaining Pb^2+^ and OH^−^ species were located, in agreement with the electron density distribution revealed by XRD, close to the wall of the framework.

### Vibrational density of states (VDOS) calculations

Vibrational frequencies of hydrogen (H) atom in different structural environment were derived from MD simulations. The analysis of the 15 ps long equilibrated trajectory of the RT Pb-STI^19^ was performed with the program TRAVIS^41^. The total and partial VDOS was calculated for: (1) all H atoms, (2) H atoms belonging to H_2_O (H_(water)_) and H atoms belonging to OH^−^ groups (H_(hydroxyl)_) and (3) H atoms belonging to H_2_O molecules that are not bonded to Pb (distance Pb–O > 3 Å) (H_(water)_0), H atoms belonging to H_2_O molecules that are bonded to Pb (distance Pb–O < 3 Å) (H_(water)_1), H atoms belonging to OH^−^ groups that are bonded to 1 Pb (H_(hydroxyl)_1) and H atoms belonging to OH^−^ groups that are bonded to 2 Pb (H_(hydroxyl)_2). The spectrum was calculated up to wavenumber 5000 cm^−1^ and the correlation depth of the ACF (autocorrelation function) was set to 256 for the calculation of all curves.

### Infrared spectroscopy

Transmission IR spectroscopy analyses were carried out on a Bruker Tensor II spectrometer equipped with a Hyperion 3000 FT-IR microscope and an MCT detector which is cooled with liquid nitrogen. The side length of the aperture square was approximately 40 µm. Each sample and background measurement consisted of 64 scans. The resolution was set to 4 cm^−1^.

Spectra were collected for several Pb-STI crystals, placed inside a Perspex chamber. Moreover, Pb-STI crystals, with dimensions ranging from ca. 20–60 µm, were placed inside a quartz glass capillary with 100 µm diameter and a wall thickness of 10–20 µm. Spectra of several crystals, inserted in the capillary, were measured at RT. The background spectrum was measured on the empty glass capillary. Afterwards, the crystals were ex situ thermally treated: the temperature was increased from RT to 430 °C with a heating rate of 40 °C/h. Then, it was kept constant for 8 h at 430 °C, before cooling down slowly. As soon as a temperature of 250 °C was reached, the capillary with the crystals was removed from the oven and the capillary was closed immediately with wax to prevent rehydration of the crystals. After cooling down to RT, the IR spectra of several crystals were measured again. The capillary was then opened and exposed to high humidity conditions (relative humidity higher than 90%). After 15 days, IR absorption spectra of several rehydrated crystals were measured.

### X-ray absorption spectroscopy: XANES

Pb-STI crystals were gently grounded and the obtained powder was investigated by Pb-L_3_ X-ray Absorption Near-Edge Structure (XANES) spectroscopy at the Rossendorf Beamline (BM20^42^) at the European Synchrotron Radiation Facility (Grenoble, France). A double crystal monochromator mounted with a Si (111) crystal was used. For each XANES measurement, the spectra of a Pb reference foil were systematically collected for post-treatment of the energy calibration. The sample was heated using a hot gas generator from Cyberstar controlled by a Eurotherm with a precision of ± 1 °C. Data were collected in situ from 25 to 400 °C.

## Supplementary Information

Supplementary Information 1.

Supplementary Information 2.

Supplementary Information 3.

Supplementary Information 4.

Supplementary Information 5.

Supplementary Information 6.

Supplementary Information 7.

## Data Availability

The datasets generated during and/or analysed during the current study are available in the Crystallography Open Database (COD) repository (entries number: 3000391, 3000392, 300093, 300094) and/or included in the article as supplementary information files.
