# Supplementary material for: Pbx(OH)y cluster formation and anomalous thermal behaviour in STI framework-type zeolites
Source: Sci Rep. 2022 Sep 24;12:15934. doi: 10.1038/s41598-022-20317-1 (PMC9509355; doi:10.1038/s41598-022-20317-1)
Supplement: Supplementary file 6 — Supplementary Information 6. [file 41598_2022_20317_MOESM6_ESM.docx]

**Supplementary Material**

**Pb_x_(OH)_y_ cluster formation and anomalous thermal behaviour in STI framework-type zeolites**

Georgia Cametti^1^*, Diana P. Roos^1^, Damien Prieur^2,3^, Andreas C. Scheinost^2,3^, Sergey V. Churakov^1,4^

*^1^Institute of Geological Sciences, Bern University, Baltzerstrasse 1+3, 3012 Bern, Switzerland*

*^2^The Rossendorf Beamline at the European Synchrotron Radiation Facility (ESRF), Avenue des Martyrs 71, 38043 Grenoble, France*

*^3^Helmholtz Zentrum Dresden Rossendorf, Institute of Resource Ecology, Bautzner Landstrasse 400, 01328 Dresden, Germany*

*^4^Paul Scherrer Institut, Forschungstrasse 111, 5232 Villingen PSI, Switzerland*

*Corresponding author: georgia.cametti@geo.unibe.ch

**Content**

**Supplementary S1:** IR Band assignment based on VDOS

**Supplementary Figures**

**Figure S1** IR absorption spectra of Pb-STI crystals

**Figure S2** Structures obtained from SC-XRD analyses of Pb-STI at RT [19] (left) and after de- and rehydration (right) projected along [010].

**Figure S3** Average structure of the model Pb12-75-0.8W obtained from MD trajectories (a) compared with that of Pb-STI measured at 75 °C by SC-XRD (b).

**Figure S4** Average atomic coordinates obtained from MD trajectories calculated for different theoretical Pb-STI models (see also Table S3). (a) Dehydrated Pb-STI, (b) Dehydrated with water hydrolysis reaction, (c) Dehydrated with 17% of Pb^2+^ oxidized in Pb^4+^. (d) Measured Pb-L_3_ XANES spectra of Pb-STI upon heating.

**Figure S5** TGA curve of Pb-STI [19] and corresponding 1^st^ derivative (DTG)

**Supplementary Tables**

**Table S1** Crystal data and refined parameters of Pb-STI after rehydration measured at RT.

**Table S2** Cell parameters obtained from MD simulation at 75°C of a partially hydrated Pb-STI structure.

**Table S3** Results obtained from MD simulations of different structural models of Pb-STI run at 400°C.

**S1: IR Band assignment based on VDOS**

Vibrational Density of States (VDOS) curves were calculated for hydrogen atoms (H) based on the DFT-equilibrated Pb-stellerite structure at room temperature [19]. According to the theoretical calculations, vibrations of H atoms result mainly in two wavenumber regions: one peak in the range of 1500 to 1800 cm^-1^ and another peak in the range of ca. 2800 to 3800 cm^-1^ (Fig. 4). VDOS curves were calculated individually for H belonging to the OH^-^ groups that are bonded to one Pb, H belonging to the OH^-^ groups that are bonded to two Pb, H belonging to H_2_O not bonded to Pb (“free” H_2_O) and H belonging to H_2_O bonded to one Pb (Fig. 4a). The first peak between 1500 and 1800 cm^-1^ does occur only for H of H_2_O molecules but not for H of OH^-^ groups. This is plausible because the peak results from the fundamental H_2_O bending mode (Smith, 1998). O–H stretching modes (Smith, 1998) explain the second, broader peak between 2800 and 3800 cm^-1^ and occur in the calculated spectra of all individual H-groups (Fig. 4a). H of OH^-^, independently if bonded to 1 or 2 Pb atoms, vibrate exclusively at wavenumbers between 3500 and 3800 cm^-1^. In contrast, spectral features of H belonging to “free” H_2_O molecules are characterized by a flatter and broader band between 3000 and 3800 cm^-1^ (Fig. 4a). Moreover, a VDOS peak of H belonging to H_2_O bonded to 1 Pb ranges from ca. 3000 to 3800 cm^-1^ and reaches its maximum at 3600 cm^-1^.

Smith, B.C., 1998. Infrared spectral interpretation: A systematic approach. CRC press.

**Supplementary Figures**

**Fig. S1.** IR absorption spectra of several Pb-STI crystals measured inside a quartz glass capillary under different conditions: **(a)** at RT, **(b)** after an *ex situ* thermal treatment up to 430 °C, **(c)** after subsequent equilibration under high humidity conditions. Red curves correspond to crystals that are smaller in size (crystal dimensions ranging from ca. 20 to 60 µm) than those selected to collect the green curves (crystal dimensions ranging from ca. 40 to 100 µm) **(b)**. Regions in the spectrum associated to O–H vibrations are highlighted by grey areas.

~~
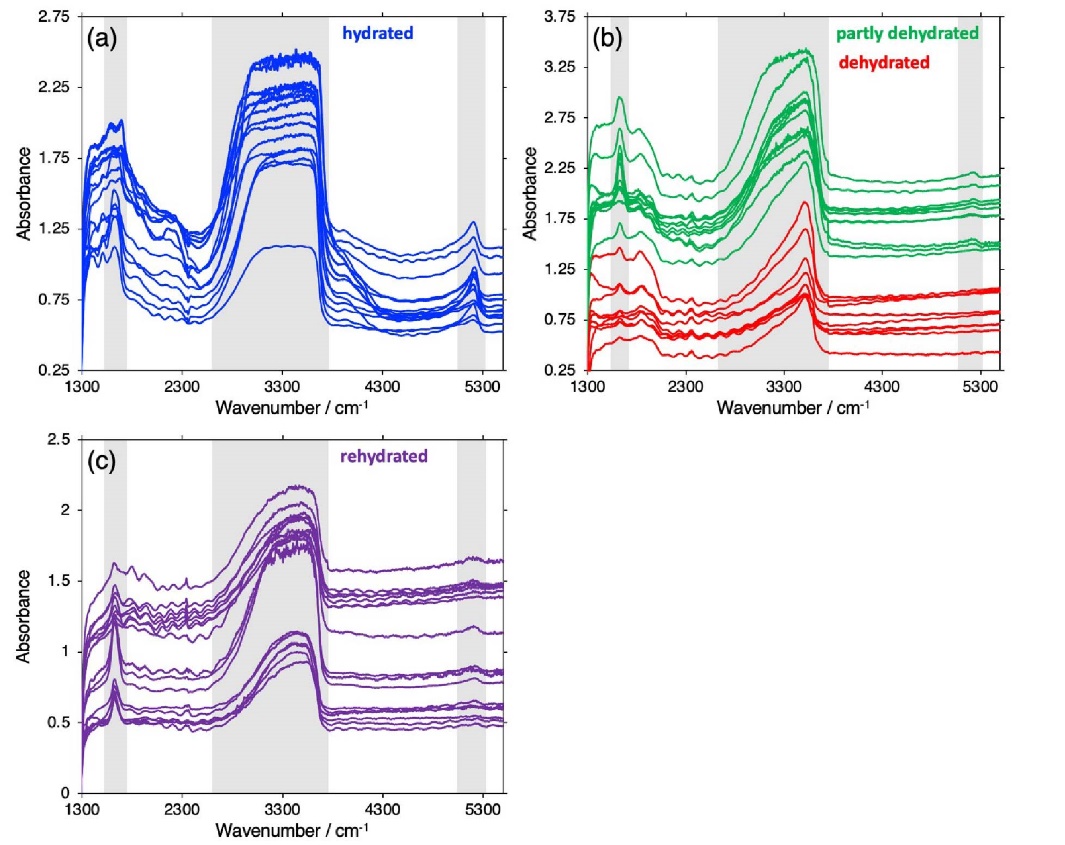
~~

**Fig. S2** Structures obtained from SC-XRD analyses of Pb-STI at RT [19] (left) and after de- and rehydration (right) projected along [010]. The framework is shown as blue tetrahedra. The EF content is as follows: partially colored dark-grey and red spheres represent Pb and O (H_2_O + OH^-^) atoms, respectively.


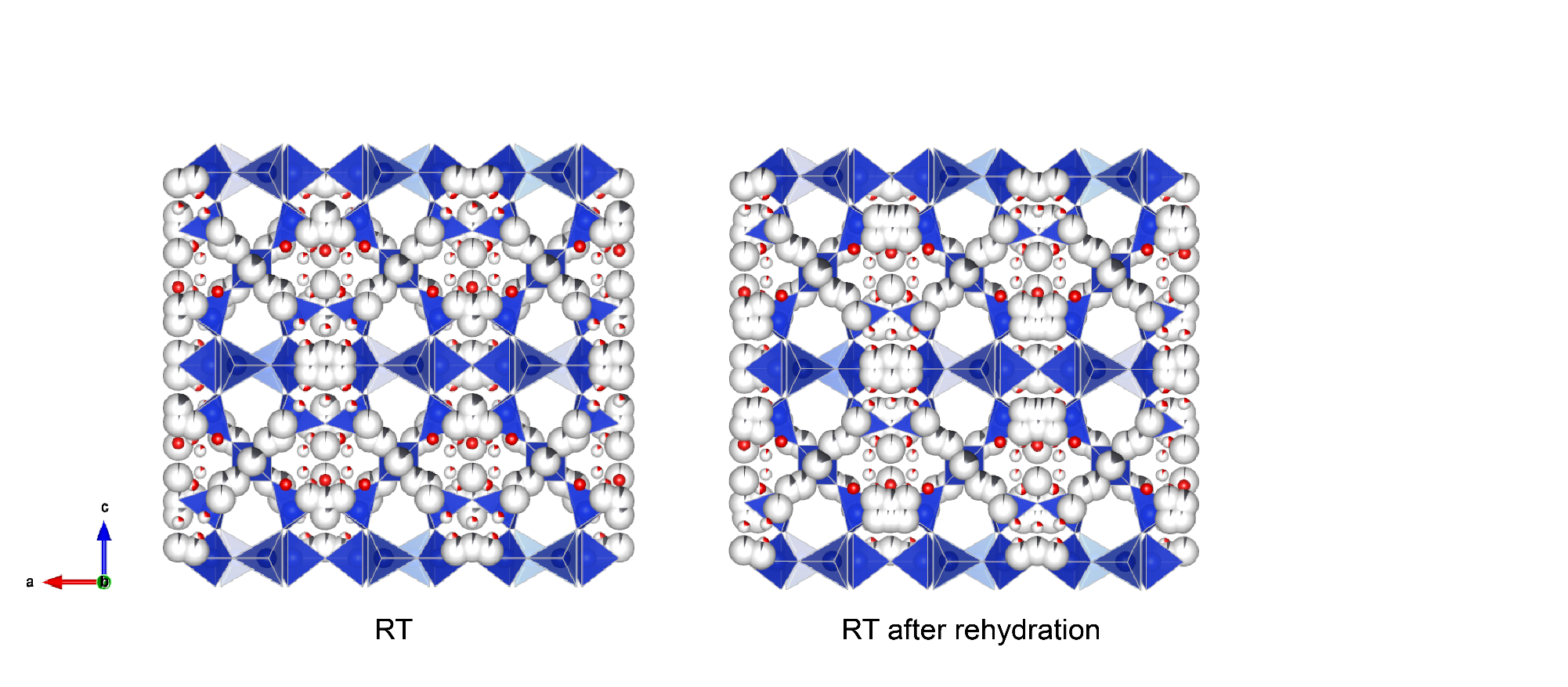


**Fig. S3** Average structure of the model Pb12-75-0.8W obtained from MD trajectories (a) compared with that of Pb-STI measured at 75 °C by SC-XRD (b).


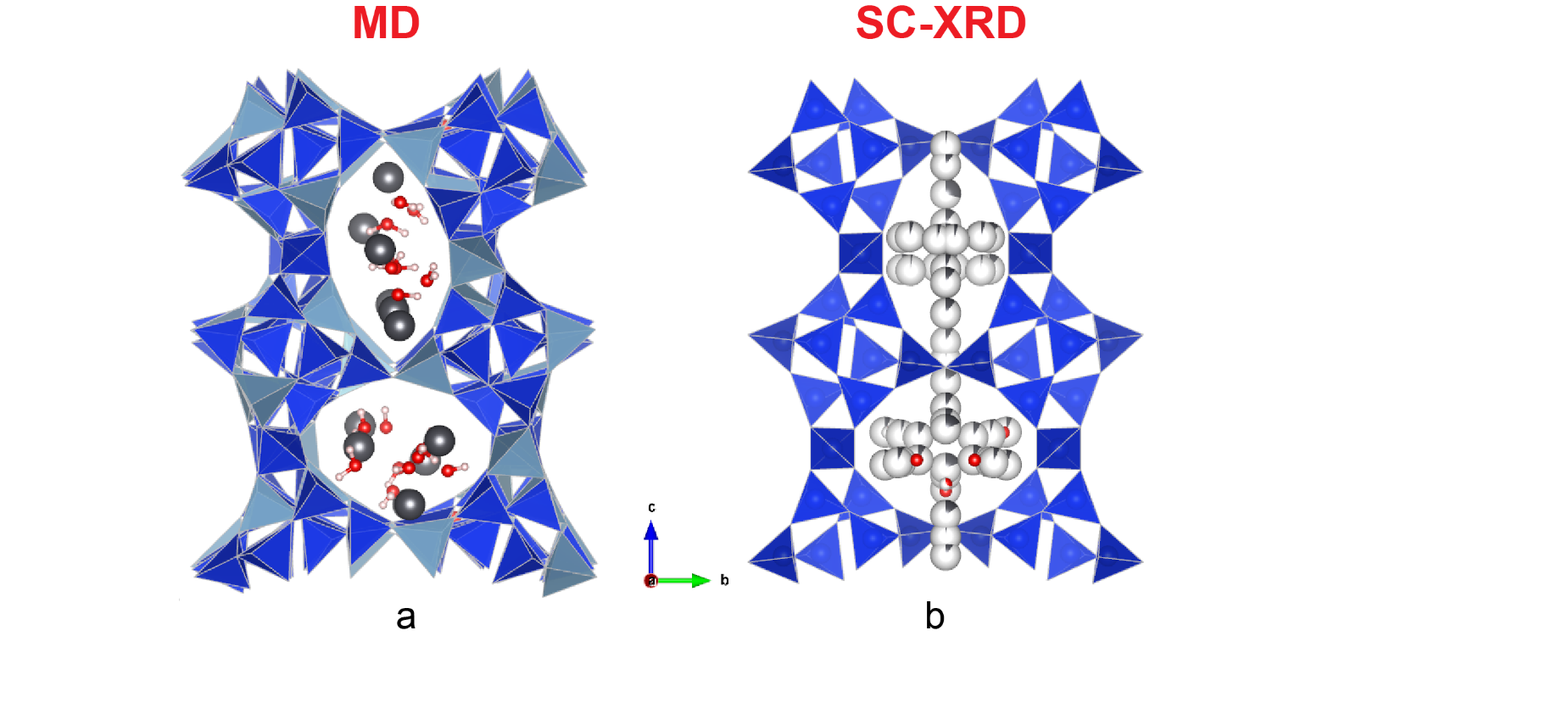


**Fig. S4** Average atomic coordinates obtained from MD trajectories calculated for different theoretical Pb-STI models (see also Table S3). (a) Dehydrated Pb-STI, (b) Dehydrated with water hydrolysis reaction, (c) Dehydrated with 17% of Pb^2+^ oxidized in Pb^4+^. (d) Measured Pb-L_3_ XANES spectra of Pb-STI upon heating.

**
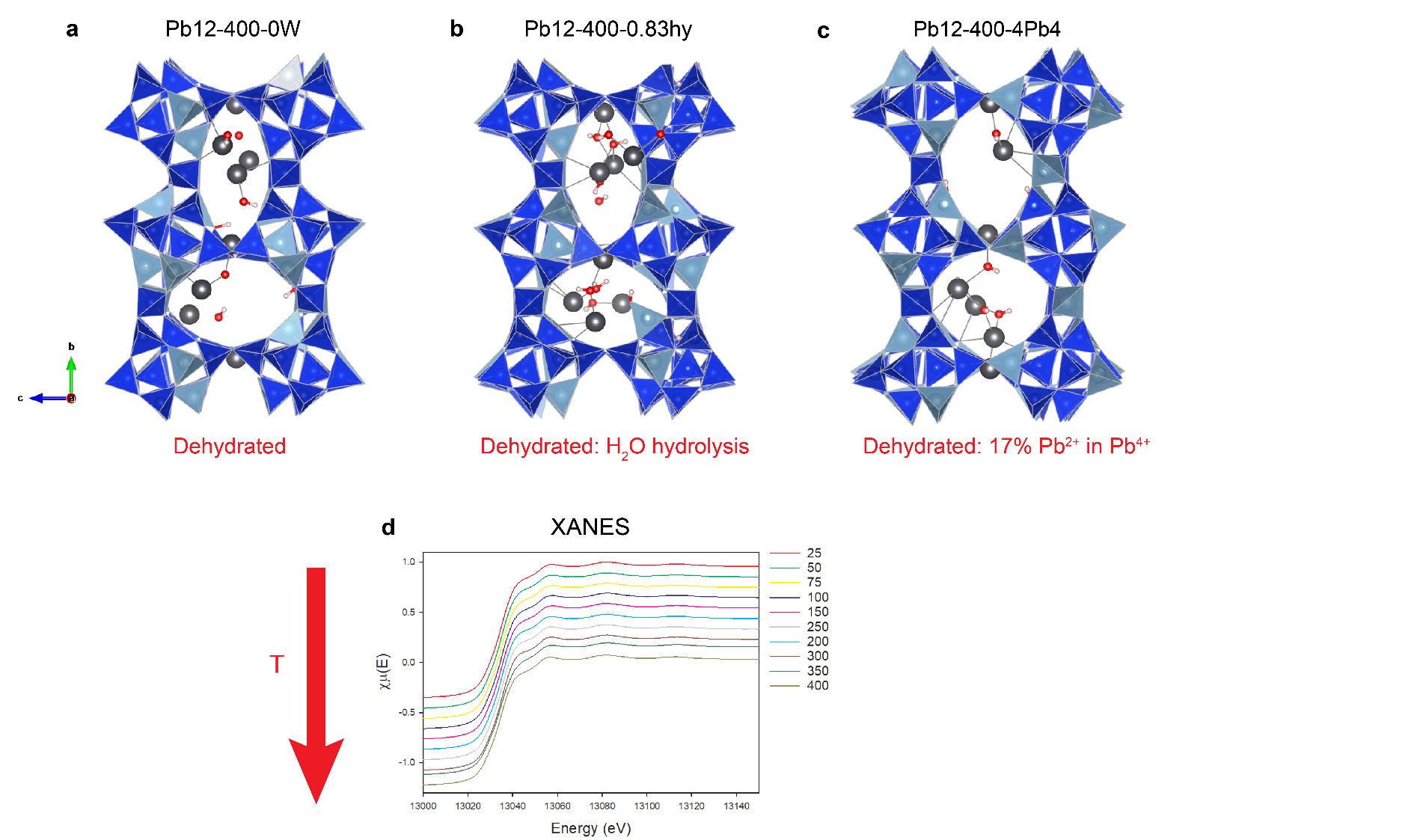
**

**Figure S5** TGA curve of Pb-STI [19] and corresponding 1^st^ derivative (DTG)


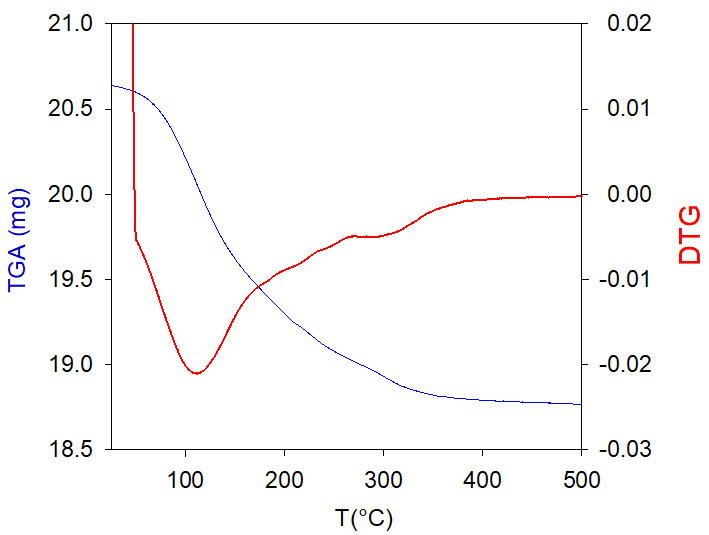


**Supplementary Tables**

**Table S1** Crystal data and refined parameters of Pb-STI after rehydration measured at RT.

| **Crystal data** | **PbSTI Rehydrated** |
| --- | --- |
| *a* (Å) | 13.6119(7) |
| *b* (Å) | 18.1833(9) |
| *c* (Å) | 17.8460(10) |
| *V* (Å^3^) | 4417.1(4) |
| *Z* | 1 |
| Space Group | *Fmmm* |
| Refined Chemical formula | Pb_12.3_(OH)_8_(Si,Al)_72_O_144_∙ 55.8H_2_O |
| Crystal size (mm) | 0.100 × 0.130 × 0.200 |
| **Intensity measurement** |  |
| Diffractometer | Bruker Apex II |
| X-ray radiation | Mo*Kα*  λ = 0.71073 Å |
| X-ray power | 50 kV, 60 mA |
| Monochromator | Graphite |
| Temperature (°C) | 25 |
| Exposure time (s) | 10 |
| Max. 2θ (°) | 51.69 |
| Index ranges | -15 ≤ h ≤ 16 |
|  | -22 ≤ *k* ≤ 16 |
|  | -21 ≤ *l* ≤ 21 |
| No. of measured reflections | 7968 |
| No. of unique reflections | 1187 |
| No. of observed reflections *I* > 2σ (I) | 848 |
| **Structure refinement** |  |
| No. of parameters used in the refinement | 133 |
| *R*(int) | 0.0592 |
| *R*(σ) | 0.0.0567 |
| GooF | 1.063 |
| *R*1, *I* >2σ (*I*) | 0.0656 |
| *R*1, all data | 0.0926 |
| *wR*2 (on *F*^2^) | 0.1834 |
| Δρ_min_ (-eÅ^-3^) close to | -0.90 W2 |
| Δρ_max_ (eÅ^-3^) close to | 0.77 C3B |
|  |  |

**Table S2** Cell parameters obtained from MD simulation at 75°C of a partially hydrated Pb-STI structure. Corresponding values of the refined structure from SC-XRD experiments are reported for comparison.

|  | **SC-XRD** | **MD** |
| --- | --- | --- |
| Reference name |  | **Pb12-75-10W** |
| Supercell content |  | Pb_24_(OH)_16_Si_112_Al_32_O_288_∙20H_2_O |
| Temperature | 75 °C | 75 °C |
| pre-equ \| equ t (ps) |  | 10 \| 14 |
| Space Group | *A*2/*m* |  |
| *a* (Å) | 13.6553(3) | 13.614 |
| *b* (Å) | 17.7611(4) | 17.859 |
| *c* (Å) | 17.5168(3) | 17.378 |
| *V* (Å^3^) | 4248.40(15) | 4223.1 |
| α | 90 | 91.4 |
| β | 90.0240(10) | 90.2 |
| γ | 90 | 90.2 |

**Table S3** Results obtained from MD simulations of different structural models of Pb-STI run at 400°C. Cell parameters of the refined structure at 400°C from SC-XRD are reported for comparison.

|  | **SC-XRD** | **MD simulations** | | |
| --- | --- | --- | --- | --- |
| Reference name |  | **M1** | **M2** | **M3** |
|  |  | Pb12-400-0W | Pb12-400-hy | Pb12-400-4Pb4 |
| Supercell content |  | Pb_24_(OH)_16_Si_112_Al_32_O_288_ | Pb_24_(OH)_56_H_40_Si_112_Al_32_O_288_ | Pb^2+^_20_Pb^4+^_4_(OH)_24_Si_112_Al_32_O_288_ |
| a (Å) | 13.6261(4) | 13.636 | 13.642 | 13.685 |
| b (Å) | 17.7090(5) | 17.464 | 17.418 | 18.013 |
| c (Å) | 17.9186(5) | 17.074 | 17.690 | 17.682 |
| V (Å^3^) | 4323.8(2) | 4065 | 4197 | 4356.5 |
| α | 90 | 89.9 | 89.60 | 90.14 |
| β | 90 | 89.79 | 90.18 | 89.66 |
| γ | 90 | 90.48 | 89.64 | 90.17 |
| pre-equ \| equ t (ps) |  | 10 \| 4 | 6 \| 3 | 15 \| 15 |
